# Tissue-Agnostic Targeting of Neurotrophic Tyrosine Receptor Kinase Fusions: Current Approvals and Future Directions

**DOI:** 10.3390/cancers16193395

**Published:** 2024-10-04

**Authors:** Mohamed A. Gouda, Kyaw Z. Thein, David S. Hong

**Affiliations:** 1 Department of Investigational Cancer Therapeutics, The University of Texas MD Anderson Cancer Center, Houston, TX 77030, USA; mgouda@mdanderson.org; 2 Comprehensive Cancer Centers of Nevada—Central Valley, Las Vegas, NV 89169, USA; kyaw.thein@usoncology.com

**Keywords:** NTRK, larotrectinib, entrectinib, repotrectinib, tissue-agnostic therapy

## Abstract

**Simple Summary:**

There has recently been an interest in drugs that work across the pan-cancer spectrum and show antitumor activity based on biomarkers rather than histology. In this article, we discuss one of these biomarkers, NTRK fusions, which, if present, can predict sensitivity to treatment with inhibitors of the NTRK molecular pathway. Currently, there are three drugs that have been approved by the United States Food and Drug Administration (FDA) in patients with NTRK fusions, regardless of the tumor tissue of origin. These are larotrectinib, entrectinib, and repotrectinib. Herein, we discuss what NTRK fusions are and how to detect them, alongside data on each drug, from a clinical perspective.

**Abstract:**

*NTRK* fusions are oncogenic drivers for multiple tumor types. Therefore, the development of selective tropomyosin receptor kinase (TRK) inhibitors, including larotrectinib and entrectinib, has been transformative in the context of clinical management, given the high rates of responses to these drugs, including intracranial responses in patients with brain metastases. Given their promising activity in pan-cancer cohorts, larotrectinib and entrectinib received U.S. Food and Drug Administration (FDA) and European Medicines Agency (EMA) approval for tissue-agnostic indications in patients with advanced solid tumors harboring *NTRK* fusions. The safety profiles for both drugs are quite manageable, although neurotoxicity driven by the on-target inhibition of normal *NTRK* can be a concern. Also, on- and off-target resistance mechanisms can arise during therapy with TRK inhibitors, but they can be addressed with the use of combination therapy and next-generation TRK inhibitors. More recently, the FDA approved the use of repotrectinib, a second-generation TRK inhibitor, in patients with NTRK fusions, based on data suggesting clinical efficacy and safety, which could offer another tool for the treatment of *NTRK*-altered cancers. In this review, we summarize the current evidence related to the use of TRK inhibitors in the tissue-agnostic setting. We also elaborate on the safety profiles and resistance mechanisms from a practical perspective.

## 1. Introduction

The use of tissue-agnostic, molecularly driven therapies has ushered in a paradigm change in the clinical management of cancer. These therapies can be used for the treatment of tumors with specific molecular alterations, regardless of the cancer’s site of origin, with encouraging data regarding their use in cases with different molecular targets [1,2]. While the concept seems appealing in the context of precision oncology, there are several issues acknowledged, including the availability and feasibility of performing the necessary tests outside of large academic centers and the turnaround time, which can sometimes be considerably long. However, there is also an element related to the awareness of the practicing oncologist, which could be potentially addressable [3].

Among genomic alterations of interest for tissue-agnostic therapy, NTRK fusions emerge as a possible target [1,4,5]. The tropomyosin receptor kinase (TRK) inhibitors larotrectinib and entrectinib were among the earliest drugs of this kind to receive approval from the U.S. Food and Drug Administration (FDA) for tissue-agnostic indications [6,7,8]. This was a notable milestone in the field of cancer management and opened new avenues for the research and development of similar therapies. More recently, the second-generation TRK inhibitor repotrectinib also received accelerated FDA approval for the management of patients with NTRK fusions [9]. Such innovative therapies have the potential to transform cancer treatment, offering hope to patients who previously had limited treatment options. In this review, we describe the practical application of TRK inhibition from a tissue-agnostic perspective, highlighting the challenges in clinical application, possible resistance mechanisms, and emerging second-generation TRK inhibitors.

## 2. *NTRK* Fusions

**Figure 1:**
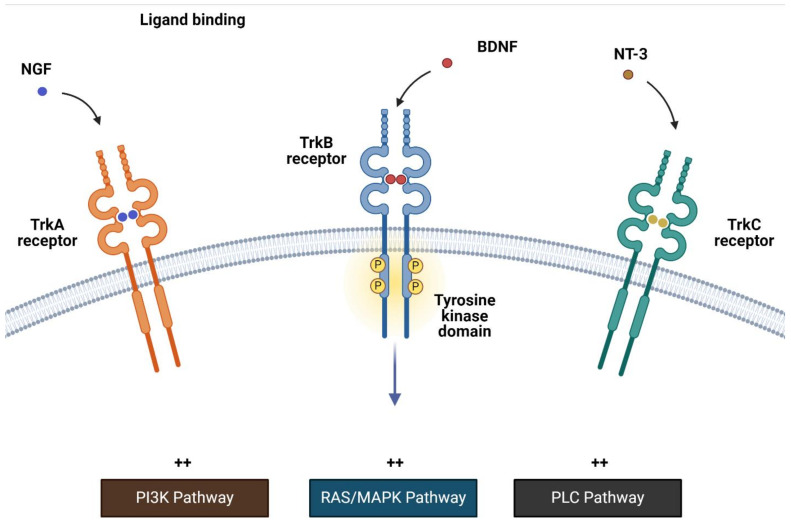
TRK receptors and signaling.

The NTRK family of genes includes *NTRK1*, *NTRK2*, and *NTRK3*, which code for receptors TRK-A, TRK-B, and TRK-C, respectively. These receptors have extracellular ligand binding, transmembrane, and intracellular kinase domains. Ligand–receptor binding of the nerve growth factor, brain-derived neurotrophic factor/neurotrophin 4, and neurotrophin 3 to TRK-A, TRK-B, and TRK-C activates a downstream signaling cascade that is essential for multiple physiological processes, including cell proliferation and survival (Figure 1) [10].

*NTRK* fusions result from chromosomal rearrangements between the C-terminal domain of *NTRK1*, *NTRK2*, or *NTRK3* and the 5′ region of a partner gene. Such rearrangements are oncogenic drivers for multiple tumor types, resulting in the uncontrolled ligand-independent activation of the TRK pathway [5,10,11]. Although consistently rare, the prevalence of *NTRK* fusions varies between different studies [1,12,13,14]. For example, in one study analyzing data from 25,792 patient samples profiled for structural variants, the authors reported rearrangements in 1.6% of patients in a pan-cancer cohort, with the highest frequencies observed in patients with thyroid cancer (17.2% of all patients with thyroid cancer) and those with salivary gland cancer (15.3% of all patients with salivary gland cancer) [1]. Also, in a systematic review of 160 studies, the authors reported prevalence rates for *NTRK* fusions of 0.03% to 0.70%. However, heterogeneity among the reviewed studies and ethnic variations in patients’ genetic profiles in the context of studies from different countries are matters of concern [12]. Furthermore, in a retrospective real-world analysis of patients in the Veterans Affairs National Precision Oncology Program, the prevalence rate for *NTRK* fusions was 0.12% in a pan-cancer cohort [15], although technological challenges were cited as a possible explanation for this low rate [16].

The authors of the study described above, reporting a pan-cancer prevalence rate of 1.6%, highlighted that the detection rate decreased to 0.2% when the analysis included all 168,423 samples within the Project GENIE database and was not limited to samples profiled for structural variants, highlighting a possible impact of the used assay’s sensitivity on these prevalence rates [1]. Notably, the frequency of *NTRK* fusions in some rare cancer types is much higher than these pan-cancer rates, possibly indicating that these cancer types are enriched in *NTRK* fusions. In fact, such fusions are considered diagnostic for some tumor types, such as mammary analogue secretory carcinoma, for which the *NTRK* fusion prevalence rate can be higher than 95% [10,17]. In other cancer types, *NTRK* association may be a distinct diagnostic entity. For example, patients with *NTRK*-positive colorectal cancer may have higher tumor mutation burdens and greater microsatellite instability than patients who are molecularly unstratified and have colorectal cancer [18].

## 3. Detection of *NTRK* as a Biomarker

Different diagnostic technologies may detect *NTRK* fusions for possible targetability with approved or investigational TRK inhibitors [19]. For example, the quick, inexpensive, and widely available pan-TRK immunohistochemistry technology can detect protein overexpression; however, it has debatable degrees of sensitivity and specificity and cannot detect fusion partners [20,21,22]. Because it can be used to evaluate all *NTRK* genes, pan-TRK immunohistochemistry’s plausibly can be used as a screening test prior to more confirmatory testing. However, some tumors may present TRK protein overexpression without underlying *NTRK* fusions, and some tumors without TRK protein overexpression may harbor *NTRK* fusions [19,20]. Variability in the interpretation of the cutoffs for expression positivity in the lab is a limitation for the clinical utility of immunohistochemistry [23].

Fluorescence in situ hybridization (FISH) has higher sensitivity and specificity than immunohistochemistry but has an increased assay complexity, requires a different assay for each gene, and has a longer turnaround time. Molecular testing, including real-time polymerase chain reaction and DNA- and RNA-based next-generation sequencing, can be used to detect *NTRK* fusions. However, high cost and assay heterogeneity and the lack of wide-scale applicability are potential challenges [19,24]. Additionally, the use of circulating tumor DNA samples, which is becoming more widely accepted as an alternative to tissue testing, can be technologically challenging. The European Society for Medical Oncology (ESMO) recommends testing with FISH, RT-PCR, or RNA-based sequencing for tumors known to harbor high rates of *NTRK* fusions, with possible screening via immunohistochemistry in unselected populations followed by confirmatory testing [19]. A multiparametric approach that combines histology and genomics for case triaging and efficient fusion detection has also been described [25].

Regardless of which method is ultimately selected, which may be influenced by the assay’s availability, cost, and turnaround time, patients with advanced solid tumors should be tested for *NTRK* fusions to identify possible therapeutic targets for TRK inhibitors. Heterogeneity among the testing platforms and whether discordant results affect clinical outcomes remain points of interest for further research.

## 4. Tissue-Agnostic TRKs

The approval of the *NTRK* inhibitors larotrectinib and entrectinib by the FDA was one of the earliest departures from the classic site-oriented management of cancer to a site-independent molecular-based therapeutic approach, their approval marked as a milestone in the field of precision oncology. Over the following years, multiple other molecules have received FDA approval for targeting pan-cancer molecular biomarkers [1]. More recently, the FDA approved the second-generation TRK inhibitor repotrectinib for the management of patients with *NTRK* fusions, adding to the arsenal of drugs working in that setting. Thus far, however, larotrectinib and entrectinib are the only drugs approved by the European Medicines Agency for tissue-agnostic indications (Table 1).

### 4.1. Larotrectinib

Larotrectinib (formerly known as BAY2757556, LOXO-101, and ARRY-470) is an orally bioavailable tyrosine kinase inhibitor with selective activity against TRK. Larotrectinib competitively blocks the ATP-binding site on the intracellular kinase domain of TRK receptors [26,27], leading to the cessation of aberrant signals and the regression of tumors [26,27]. The drug is currently approved for treatment in patients with advanced solid tumors and *NTRK* fusion but without resistance mutations whose disease has progressed after prior therapy or who have no satisfactory alternative treatment options [28].

Larotrectinib is available in 25 mg and 100 mg oral capsules and as an oral solution. The currently approved dose of larotrectinib is 100 mg taken orally twice daily in adult patients and 100 mg/m^2^ taken twice daily in pediatric patients, not exceeding a total dose of 100 mg twice daily [28]. Larotrectinib is metabolized primarily by hepatic cytochrome P450; therefore, drug–drug interactions can be observed with strong cytochrome P450 3A4 inducers or inhibitors. Dose adjustment may be needed in cases with toxicity or impaired Child–Pugh B or C liver dysfunction [28].

### 4.2. Entrectinib

Entrectinib (formerly known as RXDX-101 and NMS-E628) is an orally bioavailable small-molecule inhibitor with primary activity against TRKA, TRKB, TRKC, ROS1, and ALK [29,30]. Entrectinib competitively inhibits ATP for these five targets, leading to the cessation of oncogenic signals [30]. The FDA approved entrectinib for the treatment of patients with advanced or metastatic solid tumors with *NTRK* fusion who have disease progression following treatment or no satisfactory alternative therapy [31]. In addition, entrectinib was also approved for the treatment of *ROS1*-positive non-small-cell lung cancer [30].

Entrectinib is available in 100 mg and 200 mg oral capsules and 50 mg packets. The currently approved dose of entrectinib is 600 mg taken orally once daily in adult patients, whereas body surface area (BSA)-dependent dosing is approved for pediatric patients [250 mg/m^2^ once daily in patients > 1 month to ≤6 months, 300 mg/m^2^ once daily in patients > 6 months with a BSA of ≤0.5 m^2^, 200 mg (flat dose) in patients >6 months with a BSA between 0.51 and 0.8 m^2^, 300 mg (flat dose) in patients >6 months and BSA between 0.81 and 1.1 m^2^, 400 mg in patients > 6 months with a BSA between 1.11 and 1.5 m^2^, and 600 mg (flat dose) in patients > 6 months and BSA ≥ 1.51 m^2^] [31]. Entrectinib is metabolized primarily by hepatic cytochrome P450; therefore, drug–drug interactions can be observed with strong cytochrome P450 3A4 and CA5 inducers or inhibitors. Dose adjustment may be needed in cases with toxicity or possible drug–drug interactions [31].

## 5. Clinical Data

The pan-cancer activity of larotrectinib was demonstrated in a pooled analysis of three clinical trials: LOXO-TRK-14001 (NCT02122913), SCOUT (NCT02637687), and NAVIGATE (NCT02576431) [32]. LOXO-TRK-14001 was a phase 1 dose-escalation trial in patients with advanced solid tumors regardless of *NTRK* status. SCOUT was a phase 1/2 trial that enrolled pediatric patients with advanced solid tumors regardless of *NTRK* status. In both trials, *NTRK* status predicted therapeutic efficacy. NAVIGATE was a phase 2 trial that included patients with *NTRK* fusion-positive cancers. The pooled analysis of patients with *NTRK* fusions in the three trials demonstrated an objective response rate (ORR) of 75% as evaluated by an independent review committee, which was consistent across different ages and tumor types. This pooled analysis of 55 patients led to the first tissue-agnostic approval of larotrectinib by the FDA [33,34]. An updated pooled analysis of 274 adult and pediatric patients suggested that they had rapid and durable responses, with an ORR of 66%, including complete responses in 23% of the patients. The median time to a response was 1.8 months, and the median progression-free survival time was 30.8 months [35]. In patients for whom larotrectinib was the first-line therapy, the ORR was 78%, with 41% of patients having complete responses. The median time to a response was also 1.8 months, and the median progression-free survival time was 46.2 months [36]. In a post hoc analysis of 12 patients with brain metastasis and evaluable disease, 9 had responses to larotrectinib. The three remaining patients with intracranial disease that was measurable as per RECIST had a complete response, a partial response, or a stable disease [33].

Similarly, the tissue-agnostic efficacy of entrectinib in patients with *NTRK* fusions was demonstrated in a pooled analysis of five clinical trials: ALKA-372-001 (NCT02097810), STARTRK-1 (NCT02097810), STARTRK-2 (NCT02568267), STARTRK-NG (NCT02650401), and TAPISTRY (NCT04589845). In a pooled analysis of the three trials that enrolled adult patients with advanced or metastatic *NTRK* fusion-positive tumors (ALKA-372-001, STARTRK-1, and STARTRK-2), the ORR was 59%, with 7% of patients having complete responses [37]. An updated analysis of these three trials at a median follow-up duration of 25.8 months demonstrated an ORR of 61.2% and a median progression-free survival time of 13.8 months. Eleven patients had measurable intracranial disease, and they had an intracranial ORR of 63.6% [38]. A separate pooled analysis of the STARTRK-NG and TAPISTRY trials demonstrated an ORR of 70% in pediatric patients across multiple tumor types [31].

Notably, the intracranial activity of both drugs in patients with disease spread to the brain was interesting given the challenging nature of treating brain metastasis and the high prevalence of brain metastasis in patients with *NTRK* fusions. Therefore, the reported efficacy in such a context is quite promising and provides a valuable and possibly crucial therapeutic option for the management of those patients [33,38,39,40].

## 6. Side Effects

The side effects related to the use of entrectinib and larotrectinib are generally manageable. In general, the most commonly occurring side effects with larotrectinib are liver toxicities (including increases in the levels of aspartate transaminase, alanine transaminase, and alkaline phosphatase and hypoalbuminemia), hematological toxicity (including leukopenia, neutropenia, lymphopenia, and anemia), fatigue, musculoskeletal pain, abdominal pain, nausea and vomiting, diarrhea, constipation, cough, pyrexia, hypocalcemia, and dizziness [28]. Similarly, entrectinib can commonly cause fatigue, musculoskeletal pain, nausea and vomiting, diarrhea, constipation, cough, dyspnea, pyrexia, dysgeusia, dysesthesia, edema, weight gain, dizziness, cognitive impairment, and vision problems. Central nervous system toxicity and skeletal fractures are concerns for both larotrectinib and entrectinib use [28,31]. In addition, entrectinib can lead to congestive heart failure, hyperuricemia, and a prolonged QT interval [31]. In a pooled analysis of on-target adverse events for 96 patients given TRK inhibitor-based therapy, weight gain was noted in 53% of the patients and necessitated pharmacological intervention in some patients. Moreover, dizziness that was possibly but not necessarily associated with ataxia was observed in 41% of the patients. Withdrawal-related pain was also reported in 35% of the patients upon discontinuation of a TRK inhibitor [41]. Because TRK is essential for the development of the nervous system, such neurological side effects are possibly related to the inhibition of TRK in normal tissue, including neuronal signaling, and the impact of TRK inhibition on quality of life can be devastating, which is supported by observations in studies using preclinical animal models [42,43,44,45,46,47].

## 7. Resistance to First-Generation TRK Inhibitors

Despite the durable responses seen with the use of first-generation TRK inhibitors, namely larotrectinib and entrectinib, tumor progression can eventually occur secondary to the development of acquired genetic alterations [10,48,49]. On-target resistance mechanisms are usually related to mutations in the NTRK solvent front-related domain, the xDFG motif, or gatekeeper residues. Such mutations lead to conformational changes in the kinase domain, thus affecting drug binding [10]. Although not directly shown in NTRK genes, some mutations have been suggested to drive the thermal instability of mutant proteins, which could also be associated with acquired resistance [50]. Other driver alterations, such as *BRAF*, *KRAS*, and *MET* alterations, have been suggested to be off-target resistance mechanisms to bypass TRK inhibition, although data suggest that on-target resistance could be more frequent [49,51,52].

## 8. Overcoming Resistance and Future Directions

### 8.1. Next-Generation NTRK Inhibitors: Repotrectinib

Second-generation TRK inhibitors have been developed to overcome the acquired resistance of cancer to larotrectinib and entrectinib. One molecule that recently received FDA approval is repotrectinib. Repotrectinib (formerly known as TPX-0005) is an orally bioavailable next-generation small-molecule kinase inhibitor with primary activity against ROS1, TRKA, TRKB, and TRKC [53,54,55]. Recently, the FDA approved the drug for the treatment of patients with advanced or metastatic solid tumors with *NTRK* fusion who have disease progression following treatment or who have no satisfactory alternative therapy. In addition to its tissue-agnostic indication, repotrectinib is also approved in patients with locally advanced or metastatic ROS1-positive non-small-cell lung cancer [9].

Repotrectinib is available in 40 mg and 160 mg oral capsules. The currently approved dose is 160 mg taken orally once daily for 14 days followed by an increase to 160 mg twice daily. Dose adjustment may be needed in cases with toxicity or possible drug–drug interactions [9]. The side effect profile of repotrectinib overlaps with that of other TRK inhibitors, with dizziness as the most frequently reported adverse event (in nearly 62% of patients). Other commonly cited adverse events include dysgeusia, peripheral neuropathy, constipation, dyspnea, fatigue, ataxia, cognitive impairment, muscular weakness, and nausea [9,56,57].

The pan-cancer efficacy of repotrectinib was shown in the phase I/II registrational trial TRIDENT-1 (NCT03093116), which included patients with advanced solid tumors harboring *ALK*, *ROS*, or *NTRK* rearrangements. The NTRK cohort included 88 patients with locally advanced or metastatic solid tumors who were either naïve to treatment with tyrosine kinase inhibitors (TKIs) or had progressed after a prior TKI-based therapy. The ORR was 58% and 50% in TKI-naïve and TKI-pretreated patients, respectively with responses seen across different tumor types. In five patients with measurable CNS metastatic disease at the baseline (two TKI-naïve and three TKI-pretreated), a response was seen in the five patients. The 12-month PFS rate was 22% and 22% in TKI-naive and TKI-pretreated patients [9,56,57,58].

### 8.2. Next-Generation NTRK Inhibitors: Other Drugs

Similar to repotrectinib, multiple other second-generation TRK inhibitors are currently being tested in clinical trials and have shown promising results. For example, the use of selitrectinib led to an ORR of 34% in a phase 1 trial (NCT03215511) [59]. Taletrectinib (DS-6051b/AB-106) also demonstrated activity in TRK-naïve patients in one study (NCT02675491), while the results of another trial testing its use in TRK-pretreated patients pending (NCT04617054) [60]. Examples of other second-generation TRK inhibitors include SIM-1803-1A and PBI-200, which have demonstrated activity both in vitro and in vivo, but clinical trials investigating these agents (NCT04671849 [SIM1803-1A] and NCT04901806 [PBI-200]) are still ongoing (Table 2) [61].

### 8.3. Combination Therapy

In the era of targeted therapy, combination therapy may be a possible way to overcome the resistance of cancer to TRK inhibitors via the targeting of emerging off-target resistance mechanisms by a second molecule. This approach has been used in numerous anecdotal cases with multiple other targets and successfully controlled disease progression secondary to the development of bypass resistance [64,65]. In the TRK inhibition field, adding crizotinib (with activity against METy) to TRK inhibition with selitrectinib led to the control of cancer progression driven by emerging MET amplification after entrectinib use not controlled by monotherapy with selitrectinib [51].

Because *NTRK* fusion can arise as a mechanism of cancer resistance to other targeted therapies, approaches to overcoming *NTRK* fusion-based resistance can be used for resistance that develops during combined TRK inhibition-based therapy and the continued inhibition of the original target. For example, in a patient receiving selpercatinib for *RET*-mutant medullary thyroid carcinoma, the addition of larotrectinib at the time of progression to address the emergence of *NTRK* fusion in a liquid biopsy analysis led to disease control. Later, an additional *ALK* fusion developed in this patient, possibly driving disease progression secondary to selpercatinib and larotrectinib use. A switch from larotrectinib to entrectinib (which has activity against both *NTRK* and *ALK*) led to further disease control [65]. Challenges related to such a dynamic approach to clinical management include the need to continuously evaluate an evolving tumor molecular profile, which is not always feasible, and the overall feasibility of the approach for institutions other than large academic centers.

## 9. Conclusions

The tissue-agnostic approvals of TRK inhibitors for patients with *NTRK* fusions have been transformative. The exploration of next-generation molecules is ongoing and will hopefully improve clinical outcomes in patients with tumors that harbor those genomic alterations.

## Figures and Tables

**Table 1:** Currently approved TRK inhibitors.

	Larotrectinib	Entrectinib		Repotrectinib
Year of FDA Approval	2018	2019		2024 *
Other Targets		ROS1; ALK		ROS1
FDA Indication in NTRK-positive cancers	Adult and pediatric patients with solid tumors and neurotrophic receptor tyrosine kinase (NTRK) gene fusion without a known acquired resistance mutation, are metastatic, or in whom surgical resection is likely to result in severe morbidity and have no satisfactory alternative treatments or who have progressed following treatment	Adult and pediatric patients older than 1 month of age with solid tumors and neurotrophic tyrosine receptor kinase (NTRK) gene fusion, as detected by an FDA-approved test, without a known acquired resistance mutation, are metastatic, or in whom surgical resection is likely to result in severe morbidity and who have progressed following treatment or have no satisfactory alternative therapy		Adult and pediatric patients 12 years of age and older with solid tumors and neurotrophic tyrosine receptor kinase (NTRK) gene fusion, are locally advanced or metastatic, or in whom surgical resection is likely to result in severe morbidity and who have progressed following treatment or have no satisfactory alternative therapy *
Other FDA-approved indications	None	Adult patients with ROS1-positive metastatic non-small-cell lung cancer (NSCLC) as detected by an FDA-approved test		Adult patients with locally advanced or metastatic ROS1-positive non-small-cell lung cancer (NSCLC)
Dosage	Adult and pediatric patients with a body surface area (BSA) ≥ 1 m^2^: 100 mg orally twice daily Pediatric patients with a BSA < 1 m^2^: 100 mg/m^2^ orally twice daily	Adults: 600 mg orally once daily Pediatric patients:		A total of 160 mg orally once daily for 14 days followed by 160 mg twice daily
		Age	Dose based on BSA	
		>6 months	≤0.50 m^2^: 300 mg/m^2^ 0.51 to 0.80 m^2^: 200 mg 0.81 to 1.10 m^2^: 300 mg 1.11 to 1.50 m^2^: 400 mg ≥1.51 m^2^: 600 mg once daily	
		> 1 month to <=6 months	250 mg/m^2^ once daily	

* Repotrectinib was approved for ROS1-positive NSCLC in 2023.

**Table 2:** Examples of new drugs with possible activity against NTRK fusions [62,63].

Drug	Clinical Trial ID	Phase
VMD-928	NCT03556228	I
FCN-098	NCT05212987	I
PBI-200	NCT04901806	I/II
	NCT05238337	I
TY-2136	NCT05769075	I
ICP-723	NCT05745623	II
	NCT05537987	I
	NCT04685226	I/II
XZP-5955	NCT04996121	I/II
FCN-011	NCT04687423	I/II
SIM1803-1A	NCT04671849	I
